# Lessons learnt in recruiting disadvantaged families to a birth cohort study

**DOI:** 10.1186/s12912-018-0276-0

**Published:** 2018-02-26

**Authors:** Amit Arora, Narendar Manohar, Dina Bedros, Anh Phong David Hua, Steven Yu Hsiang You, Victoria Blight, Shilpi Ajwani, John Eastwood, Sameer Bhole

**Affiliations:** 1 School of Science and Health, Western Sydney University, 24.2.97 Campbelltown Campus, Locked Bag 1797, Penrith, NSW 2751 Australia; 2 Sydney Dental Hospital and Oral Health Services, Sydney Local Health District, Surry Hills, NSW Australia; 3 Discipline of Paediatrics and Child Health, Sydney Medical School, Westmead, NSW Australia; 4 Collaboration for Oral Health Outcomes Research, Translation, and Evaluation (COHORTE) Research Group, Ingham Institute for Applied Medical Research, Liverpool, NSW Australia; 5 Faculty of Dentistry, The University of Sydney, Surry Hills, NSW Australia; 6 Child and Family Health Nursing, Primary & Community Health, South Western Sydney Local Health District, Narellan, NSW Australia; 7 Department of Community Paediatrics, Sydney Local Health District, Croydon Community Health Centre, Croydon, NSW Australia; 8 Sydney Medical School, The University of Sydney, Sydney, NSW Australia; 9 School of Women’s and Children’s Health, UNSW Australia, Kensington, NSW Australia; 10 School of Medicine, Griffith University, Gold Coast, QLD Australia

**Keywords:** Oral health, Longitudinal research, Cohort study, Nurses, Children, Early childhood caries

## Abstract

**Background:**

Dental decay in early childhood can be prevented by a model based on shared care utilising members of primary care team such as Child and Family Health Nurses (CFHNs) in health promotion and early intervention. The aims of this study were to identify the facilitators and barriers faced by CFHNs in recruiting research participants from disadvantaged backgrounds to a birth cohort study in South Western Sydney, Australia.

**Methods:**

Child and Family Health Nurses recruited mothers-infants dyads (*n* = 1036) at the first post-natal home visit as part of Healthy Smiles Healthy Kids Study, an ongoing birth cohort study in South Western Sydney. The nurses (*n* = 19) were purposively selected and approached for a phone based in-depth semi-structured interview to identify the challenges faced by them during the recruitment process. Interviews were audio-recorded, subsequently transcribed verbatim and analysed by thematic analysis.

**Results:**

The nurses found the early phase of parenting was an overwhelming stage for parents as they are pre-occupied with more immediate issues such as settling and feeding a newborn. They highlighted some key time-points such as during pregnancy and/or around the time of infant teething may be more appropriate for recruiting families to dental research projects. However, they found it easier to secure the family’s attention by offering incentives, gifts and invitations for free oral health services. The use of web-based approaches and maintaining regular contact with the participants was deemed crucial for long-term research. Cultural and linguistic barriers were seen as an obstacle in recruiting ethnic minority populations and the need for cultural insiders in the research team was deemed important to resolve the challenges associated with conducting research with diverse cultures. Finally, nurses identified the importance of inter-professional collaboration to provide easier access to recruiting research participants.

**Conclusions:**

This study highlighted the need for multiple time-points and incentives to facilitate recruitment and retention of disadvantaged communities in longitudinal research. The need for cultural insiders and inter-professional collaboration in research team are important to improve research participation.

## Background

Dental caries (tooth decay) is one of the most common multifactorial chronic disease affecting children [1]. When it occurs in children aged less than 6 years, it is referred to as Early Childhood Caries (ECC) which is defined as the “presence of one or more decayed (non-cavitated or cavitated lesions), missing (due to caries), or filled tooth surfaces in any primary (baby) tooth” [2]. ECC is a serious oral health problem which is widespread in many populations across the world and especially prevalent in socially disadvantaged groups [3, 4]. The most recent Australian National Child Oral Health Survey 2012–2014 reported that over 34% of 5–6-year-olds had one or more decayed, missing (due to caries) and filled primary teeth [5]. The problem of ECC is not limited to Australia, as similar findings have also been observed internationally [6].

The effects of ECC extend beyond the primary dentition and have significant comorbidities – increased likelihood to have poor oral health in adulthood; stress on the child’s family; repeated prescription of antibiotics, severe pain, sepsis, and sleep loss; increased financial burden; poor quality of life; and often a burden on the healthcare system as in severe cases or in non-compliant children treatment under a general anaesthetic is necessary [7]. There is ample evidence to show that preventive oral health messages provide a proven health benefit [8]. The success of oral health promotion interventions require appropriate health behaviours to be established early in life; hence, there is a need to focus on preventative advice to pregnant women [9] and parents of young children [4, 10]. However, visits to an oral health professional in early childhood are often limited [11] and most visits are either to a general medical practitioner or to a Child and Family Health Nurse (CFHN) [12, 13].

In the recent years, most Australian states and territories support an early childhood oral health program that links oral health professionals with general health professionals [14, 15]. Several developments have taken place in NSW since the mid-2000s in integrating the shared care model for oral health promotion. Since 2007, early childhood oral health training has been available to all health professionals including CFHNs [12] and more recently, the Midwifery Initiated Oral Health program has been introduced in South Western Sydney and Western Sydney [9, 16]. These recent developments are major achievements in early intervention strategies and utilising the shared care model approach is prudent for improving oral health outcomes.

Longitudinal research is an important approach in uncovering potential solutions for ECC. Prospective cohort studies, although time consuming and expensive to implement, offer good scientific evidence in understanding the disease mechanisms, however, recruitment and retention of research participants may be problematic and can significantly impact the study findings. Insufficient recruitment could make a study underpowered and study sample attrition could affect the validity of the study findings. The ability of a study to establish and maintain its participants increases the validity of the study as it reduces problems associated with selection bias and non-response [17]. Numerous obstacles pose as a threat to recruitment and retention of research participants. These may include issues such as lack of cultural sensitivity towards participants, lack of trust with healthcare system, concerns of participants being a “guinea pig”, limited literacy skills of participants, and personal commitments of research participants [18–22].

The literature on challenges with recruiting and retaining research participants is primarily from the United States [18–22], with limited information from Australia, particularly South Western Sydney as this is an ethnically diverse region with high levels of social disadvantage [23]. Furthermore, birth cohort studies are rare in dentistry and to the best of our knowledge none of the longitudinal dental research projects have discussed the challenges in recruiting research participants. The aims of this study therefore, were to identify the facilitators and barriers faced by CFHNs in recruiting research participants from disadvantaged backgrounds to a birth cohort study in South Western Sydney.

## Methods

### Study background

This qualitative study is nested within an ongoing birth cohort study, ‘Healthy Smiles Healthy Kids’ (HSHK), investigating the relationship between early childhood feeding patterns, oral health and obesity among pre-school children in South Western Sydney [24]. For this project, CFHNs recruited mother-infant dyads at the first post-natal home visit at 4 to 6 weeks, as this is the primary point of community-based health professional contact for newborn children and their carers/parents [12, 13]. A total of 1500 mothers who gave birth to infants between October 2009 and February 2010 in public hospitals located under the catchment of the former Sydney South West Area Health Service (now separated as Sydney and South-Western Sydney Local Health Districts) were approached to be a part of this study. At the first post-natal visit, CFHNs explained the project to the mothers and obtained a written informed consent. If requested, the nurses were able to arrange for interpreter services for non-English speaking parents and language appropriate written materials were provided for the major ethnic groups living in this region (i.e. Vietnamese, Arabic, Assyrian, Cambodian, Cantonese, Hindi, Mandarin, and Samoan).

### Study sample

Nurse Unit Managers in the former South-Western Sydney Area Health Service were contacted to obtain details of the CFHNs who could represent all geographical sectors of South Western Sydney. The CFHNs (*n* = 21) were purposively selected and invited to participate in this qualitative study. Selection was based on ethnicity, years of experience, and geographical location. They were given an information pack that provided details of the nested study, a written consent form, and a participation information statement. All nurses initially agreed to participate but two could not be contacted for an interview despite repeated attempts and were therefore excluded. It has previously been outlined that for qualitative research with a homogenous sample such as in this study, six to eight interviews are sufficient to reach data saturation [25]. This study involved interviewing a larger number of participants (*n* = 19), with a wide variation in participant characteristics in order to enrich our data quality [26].

### In-depth interviews

In-depth semi-structured interviews were conducted by two researchers (DB and AA) to record views of CFHNs on the facilitators and barriers they encountered in recruiting research participants for the HSHK study. The interviews were phone-based as the study sample was geographically dispersed [27]. The researchers used an interview guide (Table 1) that covered topics relevant to the recruitment process. The development of interview guide was informed by a comprehensive review of the literature to identify key areas of interest [18–22]. The draft interview guide was piloted with two CFHNs. Each interview lasted about 30–45 min and we continued interviewing CFHNs until no new topics emerged i.e. data reached saturation. All interviews were audio-recorded and transcribed *verbatim*. Interview debriefing between the two researchers (DB and AA) was consistently undertaken to evaluate the data collection, summarise the main findings and prepare for subsequent interviews.

**Table 1:** Semi-Structured interview questions

• What are some facilitating factors you found in recruiting families with newborn children?
• What barriers did you find recruiting families of newborn children?
• What do you think can be done to improve the recruitment process?
• How can we motivate potential participants that are not interested to participate in the study?
• Is there anything we can change to make the program more successful, especially for people in disadvantaged areas?
• Can you explain if the study recruitment caused any problems for you?

### Data analysis

To enhance the rigour and credibility of our research, four researchers (AA, APDH, SYHY, DB) were involved in every phase of data analysis, including transcript coding, data display and interpretation. The four researchers coded the transcripts individually: two using manual coding, and two using NVivo 9 (QSR International, Cambridge, MA, USA) software, and the findings were compared. A consensus was then agreed upon between the four researchers and advice was sought from a fifth coder (NM) when disagreements arose. The codes were then examined through an iterative process and regrouped into four broad themes. A thematic analysis approach was used, as this method has been shown to be effective in “identifying, analysing and reporting patterns within qualitative data” [28].

### Ethics

Ethics approvals for this study were obtained from the former Sydney South West Area Health Service – RPAH Zone (ID number X08–0115), Liverpool Hospital, University of Sydney, and Western Sydney University. All participants signed a written consent form to be a part of this study. This research has been conducted in full accordance with the World Medical Association Declaration of Helsinki. Written consent was obtained from all study participants.

## Results

Table 2 shows the characteristics of the study participants. Most of the CFHNs were aged 40 years or older, had a postgraduate diploma or a Masters degree and had over 10 years of professional work experience. Four themes emerged from the data. These were: participant’s concern of receiving overwhelming information during early stages of parenthood, strategies to improve research participation, cultural barriers and involvement with research, and the emphasis for inter-professional collaboration.

**Table 2:** Demographic characteristics of the study participants (*n* = 19)

Characteristic	N
Age (in years)	
30–39	3
40–49	9
50+	7
Highest education qualification	
Bachelors	4
Postgraduate Diploma	9
Masters	6
Nursing experience (years)	
1–9	7
10–19	9
20–29	3

### Theme one: Overwhelming information during early stages of parenthood

The majority of nurses reported that one of the difficulties in recruiting participants was that parents felt overwhelmed with information, particularly during the first visit thus making the recruitment challenging.

*“We get around to giving them all the other information then we tell them to be part of a research study I think they’ve had enough.”* (Nurse #2)

The nurses believed that one of key reasons for families to not join research projects in early stages of parenting is due to the fact that many parents have other important issues on their minds during the first few weeks of the baby’s birth. Matters directly related to child well-being play a major significance in the early stages of parenting compared to other matters. Furthermore, the nurses were worried that they did not want to add additional stress and commitments to the families during the initial stages of parenthood.

*“They (parents) still have sleeping/settling issues or breastfeeding or bottle feeding issues and they may not necessarily be taking it in at that point.”* (Nurse #7)

*“Probably it’s not very appropriate on the first visit because we have to give them so many other things. When we first see them in the first 2 weeks they’re not ready, their head’s not really there and they can’t take in too much information.”* (Nurse #14)

They also reported that families that were undergoing difficult circumstances prioritise and focus their time on other significant issues before undertaking more obligations.

*“We only see two families a day with children from zero to 3 years of age and up to 20 percent of them have significant mental health issues. They are so overwhelmed that they want to talk about other stuff.”* (Nurse #9)

### Theme two: Strategies to improve research participation

The nurses thought that early days of infancy are tough periods to recruit families for research projects. However, they highlighted that it may be wise to recruit mothers during pregnancy or around the time of infant teething.

*“For a dental project, we could involve families when the baby is four or five months old. That’s when the mums usually ask about teeth as the baby starts drooling and shows some signs of teething”* (Nurse #1)

The nurses highlighted that the families were more inclined to participate in research studies if there were incentives for them.

*“They (families) are keen if there is something free they are getting out of it”* (Nurse #5)

The nurses reported that incentives could include anything from free telephone helpline on health education to free goods. In particular, they reported that things that were relevant to new parents are of importance as they would enjoy receiving it.

*“Participants found out that they get health information and freebies such as sipper cup, teething ring, free toothpaste, toothbrushes and dental care - they are happy for that!”* (Nurse #8)

The nurses highlighted that strategies need to be put into place to retain participants for long-term research. In general, it was suggested that getting multiple contact details of the families was crucial for research in disadvantaged areas. It was further suggested that linking the research to their hospital records will be a useful way to retain participants.

*“You should take two numbers. Maybe even two mobile numbers as these days both parents have mobile numbers, or maybe say grandmother’s number. This way you can send them reminders and could keep in contact with them even if they move homes or change numbers.”* (Nurse #17)

*“You should link it to their medical and /or dental records. That way you could possibly keep updates if they move homes or change numbers”.* (Nurse #11)

Some nurses even suggested that sending newsletters to participants or perhaps creating a website for the research would be useful so that participants can keep themselves updated and be more involved with the project.

*“You know a lot of times research fails as it becomes tough to maintain the interest of the participants. I think sending newsletters say every 3-6 months giving them updates and/or maybe creating a website keeps them engaged”.* (Nurse #14)

### Theme three: Cultural barriers and involvement with research

The nurses found that the difficulties in recruiting participants were largely due to interpretational and cultural barriers. It was noted that the information on research should be communicated in a clear and concise manner so that it could be easily understood by participants of linguistically diverse backgrounds. The nurses recognised that the cohort study could potentially benefit a lot of families especially those from ethnic minorities.

*“The document needs to be clear, simple and short. There are quite a few people who need interpreting.”* (Nurse #13)

*“It would be good to have them in other languages… It seems to be working with a lot of Chinese or Indian families”* (Nurse #10)

Some nurses pointed out that it is important to resolve ‘cross-cultural differences’ in research projects. They believed that it might be easier to recruit and retain participants if members of the research team are from the same cultural background to that of the research participants. They believed that this might assist in gaining trust of the participants.

*“It is a new environment for them (migrants), and some of them are so reliant on their families back home. I think it is good they know the research team and if they are from similar cultural background, they may see them as more trustworthy. Not just feel that I am trying to sell anything to them!”* (Nurse #5)

A few nurses highlighted that migrants are reluctant to participate in research due to the mistrust in health care system. They felt that migrants from developing countries had bad experiences in their home country and lose the trust in the health care system.

*“They (new migrants) are unaware of the health care system in Australia. It’s very different from their home country and a lot of them believe the system is corrupt. They think they are being treated as guinea pigs.”* (Nurse #11)

The nurses also noticed a general trend during recruitment of participants for the research study. They reported that at times it became difficult for them to convince families of ethnic minority groups to be a part of the research as they didn’t realise the benefit of research.

*“Usually it takes a lot longer to convince non-English speaking families and some of my lower side families (low socio-economic) do not see the personal benefit.”* (Nurse #15)

### Theme four: The emphasis for inter-professional collaboration

The nurses recognised the issue that young children do not visit an oral health professional unless they are in pain and there was a need for oral health promotion at an early stage. The nurses were happy to collaborate in the research study as dental disease in young children affected their working lives. However, there was a strong case for the need to arrange more appropriate time to facilitate recruitment and reduce the burden on nurses. The nurses stressed the fact that they have overwhelming amount of paperwork that needs to be completed at the first post-natal visit and that sharing information about research adds more to their workload.

*“At the first visit, we (the nurses) have so much stuff to do. It is really a challenge to recruit at the first visit as we don’t get enough time to talk about everything”.* (Nurse #8)

Some nurses found that the process of recruiting parents was a lot of work, as they had to discuss the research study with the family and take an informed consent. Although this was a daunting task, they reported that good working relationship between the researchers and nurses helped them to align their interest in promoting the program more effectively.

***“****Dr X that came to our meeting, he was very convincing to me. It gave me lots of enthusiasm to keep going with it.”* (Nurse #12)

The nurses also highlighted the fact collaboration between researchers, oral health professionals, CFHNs, made the access and recruitment of the research participants easier. They suggested that future longitudinal research projects should also consider other avenues for recruiting research participants. These could include recruiting women during pregnancy, in mothers groups, through community organisations, or involving medical doctors and lactation consultants. These multiple recruitment avenues will only be possible if health professionals work collaboratively.

*"The parenting groups was quite a good audience... they're a good opportunity to get as many clients as possible"* (Nurse #9)

*“ Perhaps the program could be targeted through the GP practices and medical centres. They all turn up to the doctors at some point.”* (Nurse #16)

*“During the first six weeks of the baby, it is tough for mums. I think getting the parents involved before the child is born is a good way to involve families or may be approach community organisations for the migrants”* (Nurse #2)

Finally, the nurses suggested that it will be useful to keep them updated with the research findings as they would feel a greater sense of accomplishment and encourages future collaborative work.

## Discussion

The CFHN is an integral member of the primary health care team in Australia as they provide support and guidance to mothers of young children on a number of health related issues including oral health. This qualitative study provides insights on the facilitators and barriers faced by CFHNs in recruiting disadvantaged families to a birth cohort study in South Western Sydney. In particular, the CFHNs recognise that dental caries is major problem in disadvantaged communities and there is need for inter-professional collaboration to promote oral health in young children. While our research aimed to identify the means by which CFHNs can efficiently and effectively connect with disadvantaged families, we found several challenges associated with communicating the importance of oral health to parents, particularly in ethnic minorities.

Some research participants in the cohort study were concerned with the amount of health information they had to absorb in the first few months of the child’s birth [29–31]. This nested study reiterated that this element created difficulty for the nurses to recruit families, as some parents were at a stage where their minds were not prepared to handle the supplementary material, causing much of it to be overlooked. When using clinicians for recruitment in research projects, other researchers [32–34] experienced similar challenges to those found in our study. Reported challenges were tension between providing care for families at a crucial time and recruiting for research, clinician’s lack of time, forgetting to mention the study to participants, and not prioritising recruitment. However, using clinicians is still a commonly used approach to recruit research participants in public health research. The nurses reported that finding alternate ways to recruit families should also be considered for such research projects. Other researchers have recruited disadvantaged families early-on during pregnancy [16, 35, 36], through medical practices [37], community health clinics [38], community groups [39, 40], or kindergartens [41].

The nurses in the study reported that future research projects could possibly use web-based approaches such as websites to recruit and retain participants. This is highlighted in a recent review on the effectiveness of web-based approaches to recruit research participants [42]. The review concluded that web-based approaches such as Facebook, Twitter, and Google adverts were effective in recruiting research participants, however, there were no significant differences in retaining participants to research studies [42]. Robinson and colleagues [43, 44] reviewed strategies to retain study participants and concluded that good organisational and communication skills of the researchers, sending out study reminders, highlighting the benefits of the study to participants, effective contact and/or scheduling strategies, community involvement, reimbursements, and incentives (financial and non-financial) were key factors for minimising attrition in research. Some of these strategies were also reported in our interviews. In particular, the nurses highlighted the importance of having multiple contact details of the participants and/or linking the research with medical and/or dental records. Other researchers have reported on the use of electronic medical records in longitudinal research is beneficial [45, 46].

Research demonstrates that digital access and use among lower income and disadvantaged groups in Australia is related to a range of broader social determinants of health, such as education, income, housing tenure, and social connections [47]. This creates a digital divide whereby people from low socioeconomic backgrounds are less likely to use smartphones and have access to the internet [47]. However, according to the Australian digital inclusion index [48], this digital divide is narrowing. Further, there is conflicting evidence that demonstrates not all low income earners are digitally disadvantaged; Choi and DiNitto [49] reported that low income people used technology despite their social disadvantage and in Australia nearly nine out of 10 people own a smartphone [50].

In this study, the CFHNs perceived that participation in dental research increased when study participants were offered incentives to take part in research. The nurses found that it was easier to secure the attention of families by offering valuable oral health information and incentives such as free sipper cups, toothpaste, toothbrushes, health promotion books, home visits and free oral health services. Many studies have illustrated that the use of incentives is an effective means to improve participation as it demonstrates a respect for the participant’s time and commitment [21, 46]. Robinson and colleagues [43, 44] recently highlighted the importance of financial incentives, non-financial incentives and reimbursements for retention of research participants. Mcsweeney and colleagues [21] reported that incentives were important for acknowledging and respecting the time and effort contributed by parents and their children. However, Baxter and colleagues [17] suggested that incentives should be carefully chosen. In the HSHK study, we decided to use incentives that were deemed appropriate for the study purposes such as oral health advice leaflets, teething ring, sipper cup, toothpaste, toothbrushes, and free oral health services to maintain interest of the participants.

The nurses observed that cultural barriers played a significant role when recruiting participants from culturally and linguistically diverse backgrounds. It was imperative for the nurses to connect with families on a level that was respectful to cultural norms and beliefs. It was advantageous for the nurses to utilise interpreters in order to build trust with the participants at the time of recruitment. Many studies have highlighted upon the importance of eliminating potential linguistic barriers by using bilingual study personnel and translated forms [17–19, 21]. The CFHNs perceived that the ethnic minority families’ lack of trust in the health care system was as a barrier to participate in health research. The perceptions of trust and mistrust of scientific investigators, of government, and of academic institutions has been a central barrier to recruitment of minority populations, particularly African migrants [19, 21, 39].

In this study, the nurses highlighted that it may easier to recruit and retain participants if members of the research team involved in recruitment are from the same cultural background to that of the research participants. Research conducted by Lee and colleagues [51] noted that communicating in native language of the study participants demonstrated respect from the study team and ensured that study participants fully understand the research to give an informed consent. Furthermore, evidence from health and social science research highlights the importance of being a cultural insider [52, 53] as they share similar social background, culture and language to that of the local people. It is suggested that cultural insiders have better insights when describing the social and cultural characteristic of the group with whom they undertake research as they are better placed to build rapport and gain trust of the participants [54]. Some researchers have suggested the use of community leaders in recruiting participants from ethnic minority communities as a way to resolve ‘power-differences’ between the practitioner and the patient [39, 51]. Therefore, it is important that culturally competent approaches and appropriate means of communication is utilised to improve recruitment. Although cultural insiders and community leaders are crucial in research, it is imperative to note that CFHNs operate from the concept of ‘cultural safety’ that emerged in the 1980s which focuses on the patient feeling safe, respected and listened to [55]. If a cultural safety approach is used, it is not necessary to utilise recruiters who share the same cultural background as participants. It redefines the patient-practitioner relationship so that it shifts the power, responsibility, and authority to lie with the patient receiving care [56, 57].

In the current study, the nurses emphasised the importance for inter-professional collaboration for successful research recruitment. Casamassimo and colleagues have highlighted the importance of inter-disciplinary research framework for improving oral health outcomes in children [58]. In recent years, most Australian states and territories support an early childhood oral health program that links oral health professionals with general health professionals [14, 15]. Since 2007, early childhood oral health training has been available to all health professionals including CFHNs [12] and more recently, the Midwifery Initiated Oral Health program has been introduced in South Western Sydney and Western Sydney [9, 16]. Furthermore, the introduction of the Medicare Benefits Schedule Primary Care Items for Healthy Kids Checks and Child Immunisation has also promoted communication between health professionals [59]. These recent developments utilising the shared care model, are major achievements in oral health promotion.

### Strengths and limitations

This study had a number of strengths that are worth reporting. Firstly, we used a qualitative approach to obtain perception of CFHN’s on recruitment of disadvantaged families to a longitudinal research project. The flexibility of the research design gives an opportunity for further investigation if required and fosters simultaneous data collection and analysis [26]. Secondly, the study had a high response rate thus achieving a 90% response rate. A sample of 19 research participants was enough to reach data saturation, that is all the dimensions of interest were explored and no new information would have been collected from interviewing more participants [60]. A potential limitation of this study was that the interviews were limited to the CFHNs in South Western Sydney; therefore, the findings may not be generalisable to all of New South Wales or Australia.

### Implications

Dental decay is one of the most common chronic childhood diseases. The results of this qualitative study reinforce the importance of a model of shared care involving members of the primary care team such as CFHN in health promotion and early intervention for preventing ECC. Recruiting disadvantaged families to longitudinal research projects is often difficult and so involving CHFNs at this stage might be advantageous since mothers are more receptive to their advice. This study highlighted that participant recruitment for research projects need to be aimed at appropriate time-points with the use of incentives. Further, web-based approaches aimed at participant recruitment were identified by CFHNs may be more innovative and effective; and regular contact with disadvantaged families another possible strategy for maximising retention. If we are to decrease health disparities among disadvantaged populations in Australia, we must find plausible solutions for dealing with the “trust” element, which in essence, is a key barrier in research participation. Gaining the trust of the culturally and linguistically diverse population groups may be possible by including cultural insiders in the research team.

## Conclusions

The CHFNs found the early phase of parenting was an overwhelming stage for parents as they are pre-occupied with more immediate issues such as settling and feeding a newborn. Other time-points such as during pregnancy and/or around the time of infant teething may be more appropriate for recruiting families to dental research projects. However, they found it easier to secure the family’s attention by offering incentives, gifts and invitations for free oral health services. The use of web-based approaches and maintaining regular contact with the participants were identified as possible strategies for continual engagement with participants. Cultural and linguistic barriers were seen as an obstacle in recruiting ethnic minority populations. However, the need for cultural insiders in the research team was deemed important to resolve the challenges associated with conducting research with diverse cultures. Finally, nurses identified the importance of inter-professional collaboration to provide easier access to recruiting research participants.

## Abbreviations

CFHN: Child and Family Health Nurses
HSHK: Healthy Smiles Healthy Kids
NSW: New South Wales
